# Extracts of *Renealmia alpinia* (Rottb.) MAAS Protect against Lethality and Systemic Hemorrhage Induced by *Bothrops asper* Venom: Insights from a Model with Extract Administration before Venom Injection

**DOI:** 10.3390/toxins7051532

**Published:** 2015-04-30

**Authors:** Arley Camilo Patiño, Juan Carlos Quintana, José María Gutiérrez, Alexandra Rucavado, Dora María Benjumea, Jaime Andrés Pereañez

**Affiliations:** 1 Food and Pharmaceutical Sciences Faculty, Pharmacy Department, University of Antioquia UdeA, Street 70 N° 52-21, Medellín 050010, Colombia; E-Mails: doraben2000@gmail.com (D.M.B.); andrespj20@gmail.com (J.A.P.); 2 Ophidism/Scorpionism Program, Food and Pharmaceutical Sciences Faculty, University of Antioquia UdeA, Street 70 N° 52-21, Medellín 050010, Colombia; 3 School of Medicine, Universidad Cooperativa de Colombia, Sede Medellín, Street 50 A N° 41-20, Medellín 050010, Colombia; E-Mail: juan.quintanac@ucc.edu.co; 4 Clodomiro Picado Institute, Microbiology Faculty, University of Costa Rica, Dulce Nombre, Vázquez de Coronado, San José 11103, Costa Rica; E-Mails: jose.gutierrez@ucr.ac.cr (J.M.G.); alexandra.rucavado@ucr.ac.cr (A.R.)

**Keywords:** *Renealmia alpinia*, snake venoms, metalloproteinase, serine proteinase, anti-venom activity, micropropagated plants

## Abstract

*Renealmia alpinia* (Rottb.) MAAS, obtained by micropropagation (*in vitro*) and wild forms have previously been shown to inhibit some toxic activities of *Bothrops asper* snake venom if preincubated before injection. In this study, assays were performed in a murine model in which extracts were administered for three days before venom injection. *R. alpinia* extracts inhibited lethal activity of *B. asper* venom injected by intraperitoneal route. Median Effective Dose (ED_50_) values were 36.6 ± 3.2 mg/kg and 31.7 ± 5.4 mg/kg (*p* > 0.05) for *R. alpinia* wild and *in vitro* extracts, respectively. At a dose of 75 mg/kg, both extracts totally inhibited the lethal activity of the venom. Moreover, this dose prolonged survival time of mice receiving a lethal dose of venom by the intravenous route. At 75 mg/kg, both extracts of *R. alpinia* reduced the extent of venom-induced pulmonary hemorrhage by 48.0% *(in vitro* extract) and 34.7% (wild extract), in agreement with histological observations of lung tissue. *R. alpinia* extracts also inhibited hemorrhage in heart and kidneys, as evidenced by a decrease in mg of hemoglobin/g of organ. These results suggest the possibility of using *R. alpinia* as a prophylactic agent in snakebite, a hypothesis that needs to be further explored.

## 1. Introduction

Envenomation by snakebites is a relevant public health issue in many regions of the world, particularly in tropical and subtropical countries of Africa, Asia, Latin America and Oceania [1]. In the case of snakes of the family *Viperidae*, which inflict the vast majority of accidents in the Americas, the pathophysiology of envenomation includes both local and systemic manifestations associated with hemorrhage, necrosis, edema, hypovolemia, nephrotoxicity, coagulopathy and cardiovascular shock [2]. This complex clinical picture is the result of the action of various venom components, predominantly proteinases metallo and serine proteinases, phospholipases A_2_, C-type lectin-like proteins, and other minor components [3].

*Bothrops asper* is responsible for 50%–80% of snakebites, and 60%–90% of deaths secondary to snakebites in Central America and northern South America [4]. Envenoming by this species induces marked local tissue damage that includes pain, edema, hemorrhage, blisters, dermonecrosis and myonecrosis [4,5]. On the other hand, the clinical manifestations of systemic alterations induced by *B. asper* venom include bleeding, coagulopathy, hypotension, hemodynamic alterations, pulmonary edema, and acute renal failure. In addition, other less common effects might occur, such as intravascular hemolysis, acute myocardial damage and, in severe cases not treated timely with antivenom, multiple organ failure and death [4,5].

The therapy for snakebite envenomations has been based on the intravenous administration of antivenoms [6]. However, it has been demonstrated that current therapy for snakebite has a limited efficacy against the local tissue damaging activities of venoms [7]. In addition, antivenoms are not available in all rural and distant places where most snakebites occur, a feature that has promoted the use of traditional medicine practices and delays the administration of specific treatment [8]. Moreover, some antivenoms induce early adverse reactions (EARs) in a high proportion of patients and some of them require cold chain for storage and transportation, a difficult task in many rural areas [8]. Thus, it is important to search for novel venom inhibitors, either synthetic or natural, that would complement the action of antivenoms.

Medicinal plants represent a vital source of novel bioactive compounds with several pharmacological activities that have contributed directly in the search of alternatives against ophidian envenomation or as a complement to conventional antivenom therapy [9]. *Renealmia alpinia* (Rottb.) MAAS (*Zingiberaceae*), known as *guaiporé*, *pintura negra*, *jazmín de monte*, *matandrea o achira de monte* [10,11,12], has been used in the traditional medicine of Colombia to treat snakebites [13]. In addition, this plant has been effective in experimental models to neutralize edema-forming, hemorrhagic, lethal, and defibrinating activities of *Bothrops asper* venom when incubated with the venom prior to injection [14,15,16]. In order to increase the productivity and homogeneity of *R. alpinia* extract, our group carried out a study with micropropagation of this plant, to obtain enough plant material, which would not be possible to achieve with traditional methods [17]. Moreover, extracts from roots and leaves of this *in vitro* grown plant inhibited the proteolytic, coagulant, and indirect-hemolytic activities of *B. asper* venom [18]. Additionally, *R. alpinia* rhizomes extract neutralized the edema-forming activity of *B. asper* venom [14]. On the other hand, Gomez-Betancur *et al.* [19] isolated a flavanone (pinostrobin) from the leaf extract of *R. alpinia*, this compound inhibited the carrageenan-induced edema.

One scenario that has received little attention regarding the potential of natural products as antagonists of venom components is the administration of extracts prior to envenomation, as a model of ingesting plant extracts in conditions of high risk of snake bites. In this work, extracts of *R. alpinia* obtained by micropropagation (*in vitro*) and wild forms were tested and compared for their capacity to inhibit lethal and hemorrhagic activities of *B. asper* venom. Results indicate that administration of these extracts during three days before venom injection exerts a significant protection in mice.

## 2. Results

### 2.1. Inhibition of Lethal Activity

**Figure 1:**
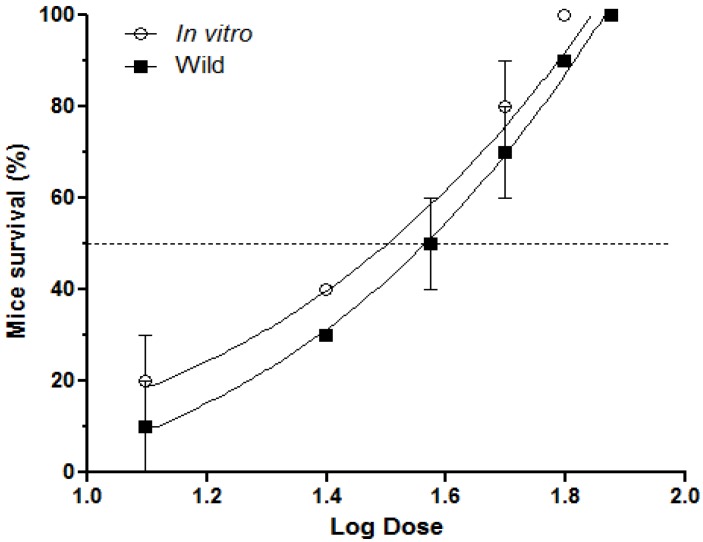
Inhibition of lethal activity induced by *B. asper* venom. During three days, groups of five mice received an intraperitoneal (i.p.) injection of either *R. alpinia* wild or *in vitro* extracts. At the fourth day, all groups were injected by i.p. route with of 1.5 × LD_50_s *B. asper* venom, and deaths were recorded during 48 h. Results are shown as mean ± SEM, *n* = 5.

*R. alpinia* extracts inhibited, in a dose-dependent manner, the lethal activity induced by 1.5 × LD_50_s*B. asper* venom (Figure 1). Both extracts totally inhibited the lethal activity of *B. asper* venom at 75 mg/kg. Moreover, at all doses used, *R. alpinia* wild and *in vitro* extracts protected mice in a comparable way (*p* > 0.05). ED_50_ values were 36.6 ± 3.2 mg/kg and 31.7 ± 5.4 mg/kg (*p* > 0.05) for *R. alpinia* wild and *in vitro* extracts, respectively. *R. alpinia* extracts were not lethal in mice at all doses tested.

On the other hand, in the assay involving pretreatment with the extracts followed by intravenous (i.v.) injection of a lethal dose of venom, there was no protection at 24 h, since all envenomed mice died. However, there was a notorious delay in the time of death in mice receiving the extracts. Mice injected with venom alone survived only 2.25 h. In contrast, animals receiving the extracts (75 mg/kg) and then venom survived 5.17 h (*in vitro* extract) and 3.83 h (wild extract) (*p* < 0.01).

### 2.2. Inhibition of Pulmonary Hemorrhage

The minimum pulmonary hemorrhagic dose (MPHD) of *B. asper* venom was 30 μg. In the inhibition assay we decided to test a dose of 40 μg venom, in order to provoke a conspicuous effect. *B. asper* venom induced a total hemorrhagic diameter of 7.5 ± 0.25 mm, when adding all the hemorrhagic spots in the surface of the lungs. In mice treated with 75 mg/kg of extracts, the sums of diameters of hemorrhagic lesions were 3.9 ± 0.19 mm and 4.9 ± 0.23, for *R. alpinia in vitro* and wild extracts, respectively (*p* < 0.05 when compared with venom alone). This represents an inhibition of pulmonary hemorrhage of 48% and 35%, respectively.

### 2.3. Histological Analysis

**Figure 2:**
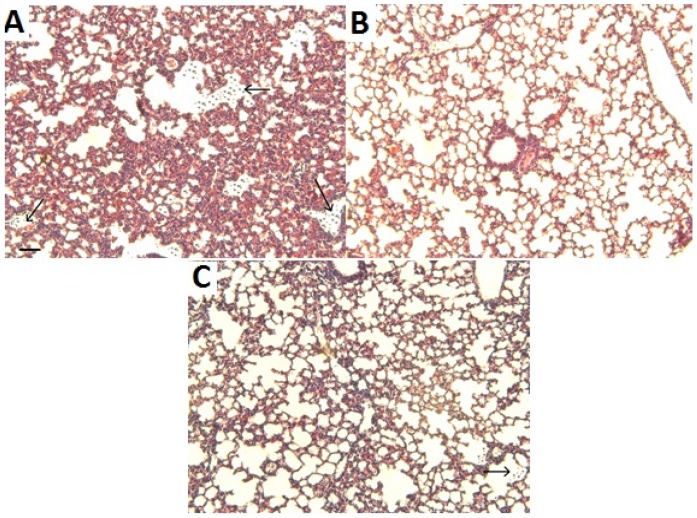
Light micrographs of sections of pulmonary tissue from mice injected i.v. with 40 μg of *B. asper* venom (**A**) or pre-treated with 75 mg/kg of *R. alpinia in vitro* extract (**B**), or wild extract (**C**), and then injected with venom. Overt hemorrhage and congestion is evident in (**A**), with erythrocytes present in the alveolar spaces (arrows), whereas the extent of extravasation is much lower in samples from mice pretreated with the plant extracts and then receiving venom (**B** and **C**). Bar corresponds to 100 μm.

Histological analysis revealed widespread hemorrhage and congestion in pulmonary tissue of mice injected with venom alone with abundant erythrocytes in the alveolar spaces (Figure 2A). In contrast, histological observations of reddish areas in lungs of mice treated with *R. alpinia* extracts before envenomation showed less extravasation than in sections from mice injected with venom alone (Figure 2B,C).

### 2.4. Inhibition of Venom-Induced Hemorrhage in Heart and Kidneys

**Figure 3:**
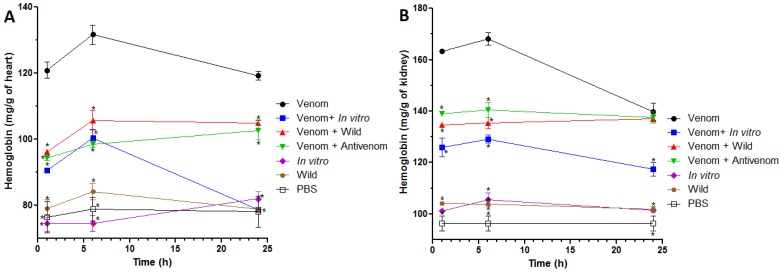
Inhibition, by plant extracts, of venom-induced hemorrhage in heart and kidneys. Mice were pre-treated with *R. alpinia in vitro* or wild extracts, and then injected with *B. asper* venom by the s.c. route. At 1, 6 and 24 h, mice were sacrificed, heart (**A**) and kidneys (**B**) were dissected, and hemoglobin (mg/g of organ) was quantified and compared with organs of animals that received venom alone or venom + antivenom. Results are shown as mean ± SEM, *n* = 4.

We also evaluated the inhibition of venom-induced systemic hemorrhage by quantifying mg of hemoglobin/g of organ in homogenates of kidneys and heart after subcutaneous (s.c.) injection of venom and pretreatment with the extracts. In both cases, venom induced its highest effect 6 h after injection. At all times, mice treated with either antivenom, *in vitro* or wild extracts showed a partial inhibition of hemorrhage, as compared with mice injected with venom alone (*p* < 0.05) (Figure 3A), although there were differences in the inhibitory activity of these three treatments. Regarding inhibition of hemorrhage in the kidneys, at 1 and 6 h, antivenom and the two plant extracts induced a significant reduction of this effect, with differences between treatments (*p* < 0.05) (Figure 3B). However, at 24 h, only *in vitro* extract exhibited significant differences when compared with venom (*p* < 0.05). The quantification of hemoglobin in heart and kidneys, after control treatments with *in vitro* and wild extracts alone was similar to that induced by PBS alone (*p* > 0.05).

## 3. Discussion

Our observations show that pretreatment of mice with *in vitro* and wild extracts of the plant *R. alpinia* protects against toxicity, *i.e.*, lethality and systemic hemorrhage, induced by the venom of *B. asper*. These findings complement previous observations showing the ability of extracts from this plant to inhibit toxic and enzymatic activities of this venom [14,16,17,18,19,20].

Lethality is the most important consequence of snakebite envenomation. The mechanisms of venom-induced lethality vary depending on the type of venom. In the case of the majority of viperid venoms, it is likely the consequence of the combined action of several toxins, and of endogenous deleterious mechanisms elicited by the action of venom components in the organism. Gutierrez *et al.* [5] hypothesized that *B. asper* venom lethal activity is likely to depend on several mechanisms. In the case of i.v. injection, the following components are likely to play a role: (a) the capacity of the Basparin A, a procoagulant P-III SVMPs, to provoke the formation of abundant thrombi in pulmonary blood vessels, probably as a consequence of thrombin generation [21]. (b) The profuse hemorrhage in the lungs of mice presented rapidly after i.v. injection of BaH4, a purified hemorrhagic P-III SVMP [22]. This agrees with other studies with various hemorrhagic P-III snake venom metalloproteinases (SVMPs), from other viperid venoms, which provokes rapid systemic bleeding [23,24,25]. (c) The ability of asperase [26], a thrombin-like serine proteinase, to induce an intravascular thrombosis upon i.v. injection. On the other hand, other, as yet unidentified, toxins are likely to play a role in lethality when the venom is administered by the i.p. route [5]. It might be that a number of venom components, which by themselves are of low toxicity, contribute to the overall toxicity of the venom by synergistic and additive effects when acting in combination with other toxins in the crude venom.

Pretreatment of mice with extracts of *R. alpinia* by the i.p. route resulted in a protection from the lethal effect of *B. asper* venom when injected i.p. at a dose corresponding to 1.5 × LD_50_s. Nevertheless, the inhibitory potential of these extracts was more limited when venom was administered by the i.v. route. This difference in inhibitory potential might have two explanations: (a) it might be that secondary metabolites of the extract, when injected i.p., accumulate in the peritoneal cavity. Hence, when venom is injected by this route, these metabolites would directly react with the venom, thus facilitating inhibition. Such direct interaction between venom and extract would occur to a much lower extent in the bloodstream after i.v. injection of venom. (b) It might be that the toxins responsible for lethality differ depending on the route. When administered i.v., it is highly likely that coagulant and procoagulant toxins play a protagonic role in lethality, through intravascular thrombosis, whereas such components are not likely to play a key role when injected i.p., owing to their slow systemic absorption. Thus, the rapid inhibition of these coagulant components in the circulation is very difficult to achieve by the plant extracts.

Systemic hemorrhage is one of the most serious consequences of envenomations by *B. asper*, resulting in severe blood loss leading to cardiovascular collapse [2,4]. Systemic hemorrhage is due to the action of hemorrhagic metalloproteinases, particularly P-III SVMPs which present various properties that make them more hemorrhagic than P-I SVMPs, such as: (a) presence of exosites in the disintegrin-like and cysteine-rich domains, enabling them to target key sites in the microvasculature [27,28]. (b) The ability to inhibit platelet aggregation [29]. (c) Resistance to the inhibitory action of α2-macroglobulin [28,30]. In addition, the effects of serine proteinases and SVMPs in hemostasis further contribute to systemic bleeding [5,31].

*R. alpinia* extracts partially inhibited venom-induced hemorrhage in lungs, kidneys and heart. These findings suggest that the extracts of this plant contain secondary metabolites able to interact, and inhibit, hemorrhagic SVMPs. In a recent study, Patiño *et al.* [20] demonstrated that *R. alpinia in vitro* and wild extracts inhibited hemorrhage, defibrin(ogen)ating, proteolytic, edema-forming, among other activities of a SVMP and thrombin-like enzymes. In that study, terpenoids, flavonoids, tannins, and coumarins were detected in both *R. alpinia* extracts. Terpenoids and coumarins have been previously reported in this species [18,32,33,34]. Some of these compounds have been reported as metal chelators [35,36]; therefore, they could chelate Zn^2+^, which is required by SVMPs for their catalytic mechanism. In the same way, other compounds such as terpenoids have been reported as SVMPs inhibitors [37], whereas coumarins have been described as serine proteinase and matrix metalloproteinase (MMP) inhibitors [38,39]. Thus, extracts of *R. alpinia* contain several types of secondary metabolites, which could mediate the inhibitory effect on hemorrhage observed in this work. The identification of these inhibitory compounds, and the study of their mechanism of inhibition, awaits further investigations. An interesting alternative possibility to explain our findings has to do with the induction of physiological changes in mice, due to the exposure to the extracts prior to venom injection, which may stimulate endogenous mechanisms involving resistance to the action of venoms.

Most studies on the effects of plant extracts on snake venoms have used a methodology based on the incubation of venom and the extract before testing *in vivo* or *in vitro* pharmacological models. Hereby we have explored an alternative model to assess inhibition, *i.e.*, by administration of the extract in mice during several days before envenomation. This method pretends to model a scenario in which extracts are ingested in situations of high risk of snakebite, thus providing protection before envenomation. The results obtained, together with previous observations on the lack of acute toxicity of extracts of *R. alpinia* [40], give support to this alternative of prophylactic use of plant extracts, a hypothesis that requires further studies using other experimental models and evaluating the chronic toxicity of these extracts.

## 4. Experimental Section

### 4.1. Venom

*Bothrops asper* venom was obtained by manual extraction of 20 specimens from the northwest region of Colombia (Antioquia and Chocó states), which are maintained in captivity at the animal house of the Universidad de Antioquia (Medellin, Colombia). Then, venom was centrifuged at 800 *g* for 15 min, and the supernatants were lyophilized and stored at −20 °C until use.

### 4.2. Animals

Swiss Webster mice (18–20 g body weight) were used for *in vivo* assays. All experiments were conducted in accordance with the guidelines of the Universidad de Antioquia Ethics Committee (expedient number 53, 16 June 2009) (Medellín, Colombia).

### 4.3. Plant Material and Preparation of Extracts of R. alpinia

The leaves of *R. alpinia* were collected in the wild of plants grown on the campus of the University of Antioquia, (1.454 meters above sea level (m.a.s.l.) voucher 107316, registration number 6456 RF. On the other hand, *R. alpinia in vitro* was obtained by the method described by Alarcón *et al.* [17]. Whole plant was used to prepare the extract. Leaves and whole plants from *R. alpinia* wild and *in vitro*, respectively, were dried at 37 °C. Then, dried and milled plant material was extracted overnight with 90% ethanol, three times. The resultant ethanol extract was concentrated at a temperature below 40 °C to a semisolid paste using a rotary evaporator (BÜCHI–124 Flawil, Switzerland). Finally, extracts were lyophilized and frozen until their use.

### 4.4. Inhibition of Lethal Activity of B. asper Venom by R. alpinia Extracts

For three days, groups of five mice received an i.p. injection of either *R. alpinia* wild or *in vitro* extract (12.5, 25.0, 37.5, 50.0, 62.5 and 75.0 mg/kg in 200 μL of 0.12 M NaCl, 0.04 M phosphate, pH 7.2 (PBS) containing 0.05% Tween. On the fourth day, all groups were injected by the i.p. route with 70 μg of *B. asper* venom in 100 μL PBS (corresponding to 1.5 × LD_50_). Control groups received either venom alone or PBS with 0.05% Tween alone. Deaths were registered within 48 h. The Median Effective Doses (ED_50_) of both extracts were calculated by Probit analysis [41]. In addition, some assays were carried out with i.p. injection of extracts for three days, as described, followed by the intravenous (i.v.) injection, via the tail vein, of 1.5 × lethal doses (105 µg) of venom on the fourth day. In this case, deaths were registered within 24 h.

### 4.5. Determination of the Minimum Pulmonary Hemorrhagic Dose

Groups of four mice (18–20 g) were injected intravenously (i.v.), via the tail vein, with various doses of *B. asper* venom dissolved in 100 μL PBS. Two and a half hours after injection, mice were sacrificed by CO_2_ inhalation. The lungs were immediately dissected out and the presence of hemorrhagic spots was assessed. The MPHD is defined as the minimum dose of venom, which induced visible hemorrhagic spots in all mice injected [25].

### 4.6. Inhibition of Venom-Induced Pulmonary Hemorrhage

For three days, groups of eight mice received an intraperitoneal (i.p.) injection of 75 mg/kg of either *R. alpinia* wild or *in vitro* extracts. On the fourth day, all groups were injected by the i.v. route, in the tail vein, with 40 μg of venom. Two and a half hours after venom injection, mice were sacrificed by CO_2_ inhalation. The lungs were immediately dissected out and the presence of hemorrhagic spots was assessed. Control groups received either venom alone or PBS with 0.05% Tween alone. The diameters of the hemorrhagic spots were quantified and the hemorrhagic activity was expressed as the sum of hemorrhagic diameters of all hemorrhagic spots; inhibition was then expressed as percentage of reduction of the sum of hemorrhagic diameters.

### 4.7. Inhibition of Hemorrhage in Heart and Kidneys

During three days, groups of four mice received an intraperitoneal (i.p.) injection of 75 mg/kg of either *R. alpinia* wild or *in vitro* extracts. On the fourth day, all groups were injected, by the subcutaneous (s.c.) route, with 60 μg of venom. In some cases, mice received an i.v. administration of 200 µL of antivenom (Antivipmyn Tri^®^, Batch B-OE-03, expiration date May 2013). One, six and twenty-four hours after venom injection, mice were sacrificed by CO_2_ inhalation. The kidneys and the heart were immediately dissected out and briefly washed in PBS to remove external blood. Then, the organs were immersed in Drabkin solution for 24 h at 4 °C, protected from the light. Subsequently, tissues were removed from the Drabkin solution and, after centrifugation; the absorbance of the supernatant at 540 nm was recorded as a quantitative index of hemoglobin present in the tissue. A standard curve of hemoglobin was used to determine the amount of hemoglobin released. Control groups received either venom alone or PBS-0.05% Tween alone or *R. alpinia* extracts (75 mg/kg). Results were expressed as mg hemoglobin/g of organ).

### 4.8. Histological Studies

For three days, groups of eight mice received an intraperitoneal (i.p.) injection of 75 mg/kg of either *R. alpinia* wild or *in vitro* extracts. On the fourth day, all groups were injected by i.v. route with 40 μg of venom. Control groups received either venom alone, PBS–0.05% Tween alone or R. *alpinia* extracts (75 mg/Kg). Two and a half hours after injection, mice were sacrificed by CO_2_ inhalation. The lungs were immediately dissected out. For histological analysis, lungs were placed in 10% formaldehyde and processed routinely for embedding in paraffin. Thick sections (4–5 μm) were prepared and stained with hematoxylin-eosin for light microscopic observation.

### 4.9. Statistical Analysis

In order to assess the significance of the differences between the mean values of various experimental groups in experiments on lethality, two-way ANOVA followed by Bonferroni’s test was applied. For the analysis of results of inhibition of the hemorrhagic activity assay, two-way ANOVA followed by Bonferroni’s test was applied. In all cases, *p* < 0.05 was considered significant, and the results are shown as mean ± SEM of n indicated in each case.

## 5. Conclusions

In conclusion, our results show that *R. alpinia* wild and *in vitro* extracts partially inhibited lethal and systemic hemorrhagic activities of *B. asper* venom, in a model with extract administration before venom injection. These results suggest the possibility of using *R. alpinia* as a prophylactic agent in snakebite, a hypothesis that requires further investigations. The similar inhibitory potential of extracts of plants propagated *in vitro*, as compared to wild ones, supports the view that *in vitro* propagation represents a valuable method to obtain extracts for pharmacological and phytochemical studies and for the *ex situ* conservation of genotypes with potential benefits in the development of new inhibitors against snake venom toxins.

## References

[1] Gutiérrez J.M., Williams D., Fan H.W., Warrell D.A. (2010). Snakebite envenoming from a global perspective: Towards an integrated approach. Toxicon.

[2] Warrell D.A., Campbell J.A., Lamar W.W. (2004). Snakebites in Central and South America: Epidemiology, clinical features and clinical management. The Venomous Reptiles of the Western Hemisphere.

[3] Calvete J.J. (2011). Proteomic tools against the neglected pathology of snake bite envenoming. Expert Rev. Proteomics.

[4] Otero-Patiño R. (2009). Epidemiological, clinical and therapeutic aspects of *Bothrops asper* bites. Toxicon.

[5] Gutiérrez J.M., Escalante T., Rucavado A. (2009). Experimental pathophysiology of systemic alterations induced by *Bothrops asper* snake venom. Toxicon.

[6] Bon C. (1996). The serum—Therapie was discovered 100 years ago. Toxicon.

[7] Gutiérrez J.M., León G., Rojas G., Lomonte B., Rucavado A., Chaves F. (1998). Neutralization of local tissue damage induced by *Bothrops asper* (terciopelo) snake venom. Toxicon.

[8] WHO (2007). Rabies and Envenomings: A Neglected Public Health Issue: Report of a Consultative Meeting.

[9] Soares A.M., Ticli F.K., Marcussi S., Lourenço M.V., Januário A.H., Sampaio S.V., Giglio J.R., Lomonte B., Pereira P.S. (2005). Medicinal plants with inhibitory properties against snake venoms. Curr. Med. Chem..

[10] Villalobos G. (1994). Plantas Comestibles en dos Comunidades de la Sierra Norte de Puebla: Xochitlán de Vicente Suárez y Zapotitlán de Méndez. Tesis de Licenciatura.

[11] Acero L.E. (1979). Principales Plantas Útiles de la Amazonia Colombiana.

[12] Standley P.C., Steyermark J.A. (1952). Zingiberaceae. Flora of Guatemala.

[13] Otero R., Fonnegra R., Jiménez S.L. (2000). Plantas Utilizadas Contra Mordeduras de Serpientes en Antioquia y Chocó, Colombia.

[14] Núñez V., Otero R., Barona J., Saldarriaga M., Osorio R.G., Fonnegra R. (2004). Neutralization of the edema-forming, defibrinating and coagulant effects of *Bothrops asper* venom by extracts of plants used by healers in Colombia. Braz. J. Med. Biol. Res..

[15] Otero R., Fonnegra R., Jiménez S.L., Núñez V., Evans N., Alzate S.A., García M.E., Saldarriaga M., del Valle G., Osorio R.G. (2000). Snakebites and ethnobotany in the northwest región of Colombia. Part I: Traditional use of plants. J. Ethnopharmacol..

[16] Otero R., Núñez V., Barona J., Fonnegra R., Jiménez S.L., Osorio R.G., Saldarriaga M., Díaz A. (2000). Snakebites and ethnobotany in the northwest región of Colombia. Part III: Neutralization of the haemorrhagic effect of *Bothrops atrox* venom. J. Ethnopharmacol..

[17] Alarcón J.C., Martínez D., Quintana J.C., Jiménez S.L., Díaz A., Jiménez I. (2008). Propagación *in vitro* de *Renealmia alpinia* (Rottb), planta con actividad antiofídica. Vitae.

[18] Fernández M., Ortiz W., Pereáñez J., Martínez D. (2010). Antiophidian properties evaluation of ethanol extract and fractions obtained from *Renealmia alpinia* (Rottb) Mass (*Zingiberaceae*) cultivated *in vitro*. Vitae.

[19] Gómez-Betancur I., Benjumea D., Patiño A., Jiménez N., Osorio E. (2014). Inhibition of the toxic effects of *Bothrops asper* venom by pinostrobin, a flavanone isolated from *Renealmia alpinia* (Rottb.) MAAS. J. Ethnopharmacol..

[20] Patiño A.C., Benjumea D.M., Pereañez J.A. (2013). Inhibition of venom serine proteinase and metalloproteinase activities by *Renealmia alpinia* (*Zingiberaceae*) extracts: Comparison of wild and *in vitro* propagated plants. J. Ethnopharmacol..

[21] Loría G.D., Rucavado A., Kamiguti A.S., Theakston R.D.G., Fox J.W., Alape A., Gutiérrez J.M. (2003). Characterization of “basparin A”, a prothrombin-activating metalloproteinase, from the venom of the snake *Bothrops asper* that inhibits platelet aggregation and induces defibrination and thrombosis. Arch. Biochem. Biophys..

[22] Franceschi A., Rucavado A., Mora N., Gutiérrez J.M. (2000). Purification and characterization of BaH4, a hemorrhagic metalloproteinase from the venom of the snake *Bothrops asper*. Toxicon.

[23] Anderson S.G., Ownby C.L. (1997). Systemic hemorrhage induced by proteinase H from *Crotalus adamanteus* (eastern diamondback rattlesnake) venom. Toxicon.

[24] Kamiguti A.S., Theakston R.D.G., Desmond H., Hutton R.A. (1991). Systemic haemorrhage in rats induced by a haemorrhagic fraction from *Bothrops jararaca* venom. Toxicon.

[25] Escalante T., Núñez J., Moura da Silva A.M., Rucavado A., Theakston R.D.G., Gutiérrez J.M. (2003). Pulmonary hemorrhage induced by jararhagin, a metalloproteinase from *Bothrops jararaca* venom. Toxicol. Appl. Pharmacol..

[26] Aragón F., Gubensek F., Rosenberg P. (1978). Characterization of thrombin-like proteinase from *Bothrops asper* venom. Toxins: Animal, Plant and Microbial.

[27] Fox J.W., Serrano S.M.T. (2005). Structural considerations of the snake venom metalloproteinases, key members of the M12 reprolysin family of metalloproteinases. Toxicon.

[28] Gutiérrez J.M., Rucavado A., Escalante T., Díaz C. (2005). Hemorrhage induced by snake venom metalloproteinases: Biochemical and biophysical mechanisms involved in microvessel damage. Toxicon.

[29] Kamiguti A.S. (2005). Platelets as targets of snake venom metalloproteinases. Toxicon.

[30] Kamiguti A.S., Desmond H.P., Theakston R.D.G., Hay C.R.M., Zuzel M. (1994). Ineffectiveness of the inhibition of the main haemorrhagic metalloproteinase from *Bothrops jararaca* venom by its only plasma inhibtor, a2-macroglobulin. Biochim. Biophys. Acta.

[31] Rucavado A., Soto M., Escalante T., Loría G.D., Arni R., Gutiérrez J.M. (2005). Thrombocytopenia and platelet hypoggregation induced by *Bothrops asper* snake venom: Toxins involved and their contribution to metalloproteinase-induced pulmonary hemorrhage. Thromb. Haemost..

[32] Lognay G., Marlier M., Severin M., Haugruge E., Gibon V., Trevejo E. (1991). On the characterization of some terpenes from *Renealmia alpinia* Rottb. (Maas) Oleoresin. Flavour Frag. J..

[33] Yang S.W., Zhou B.N., Malone S., Werkhoven M.C., van Troon F., Wisse J.H., Kingston D.G. (1999). A new labdane diterpenoid from *Renealmia alpinia* collected in the Suriname rainforest. J. Nat. Prod..

[34] Zhou B.N., Baj N.J., Glass T.E., Malone S., Werkhoven M.C., van Troon F., David, Wisse J.H., Kingston D.G. (1997). Bioactive labdane diterpenoids from *Renealmia alpinia* collected in the Suriname rainforest. J. Nat. Prod..

[35] Fernandez M.T., Mira M.L., Florêncio M.H., Jennings K.R. (2002). Iron and copper chelation by flavonoids: An electrospray mass spectrometry study. J. Inorg. Biochem..

[36] Mira L., Fernandez M.T., Santos M., Rocha R., Florêncio M.H., Jennings K.R. (2002). Interactions of flavonoids with iron and copper ions: A mechanism for their antioxidant activity. Free Radic. Res..

[37] Da Silva J.O., Fernandes R.S., Ticli F.K., Oliveira C.Z., Mazzi M.V., Franco J.J., Giuliatti S., Pereira P.S., Soares A.M., Sampaio S.V. (2007). Triterpenoid saponins, new metalloprotease snake venom inhibitors isolated from *Pentaclethra macroloba*. Toxicon.

[38] Pochet L., Frédérick R., Masereel B. (2004). Coumarin and isocoumarin as serine protease inhibitors. Curr. Pharm. Des..

[39] Yamazaki T., Tokiwa T. (2010). Isofraxidin, a coumarin component from *Acanthopanax senticosus*, inhibits matrix metalloproteinase-7 expression and cell invasion of human hepatoma cells. Biol. Pharm. Bull..

[40] Patiño A.C., López J., Aristizábal M., Quintana J.C., Benjumea D. (2012). Evaluation of the inhibitory effect of extracts from leaves of *Renealmia alpinia* Rottb. Maas (*Zingiberaceae*) on the venom of *Bothrops asper* (mapaná). Biomedica.

[41] Finney D.J. (1971). Probit Analysis.

